# *HMGA2* associated ceRNA-HOTAIR pathway in breast cancer patients from clinicopathological perspective

**DOI:** 10.55730/1300-0144.6027

**Published:** 2025-05-30

**Authors:** Pelin ERCOŞKUN, Deniz AĞIRBAŞLI, Mehmet VELİDEDEOĞLU, Mehmet SEVEN, Aysel KALAYCI

**Affiliations:** 1 Department of Medical Genetics, Faculty of Medicine, İstanbul University-Cerrahpaşa, İstanbul, Turkiye; 2 Department of General Surgery, Faculty of Medicine, İstanbul University-Cerrahpaşa, İstanbul, Turkiye

**Keywords:** Breast cancer, HOTAIR, *HMGA2*, noncoding RNA, ceRNA

## Abstract

**Background/aim:**

Several epigenetic alterations are involved in the development of breast cancer. The long noncoding RNA HOTAIR and related RNAs play a role in initiation of breast cancer and are promising targets for diagnostic biomarker and therapeutic studies. In this study, we aimed to investigate *HMGA2* associated ceRNA HOTAIR pathway in breast cancer patients.

**Materials and methods:**

Forty breast cancer patients and ten healthy controls were included in this study, and then patients were divided into clinicopathologic groups. After total cell-free RNA isolation, expression levels of target RNAs were analysed by Real-Time PCR. The amount of gene expression was determined according to delta-delta Ct method and change in the expression was determined using the 2^−ΔΔCt^ method.

**Results:**

HOTAIR expression was significantly higher in the study group (especially in the ER negative group) than in the control group (p value = 0.006). When patients with relapse were compared with those without relapse, *HMGA2* expression was significantly higher (p value = 0.048). There was a significant increase in miR-20a-5p expression (p value = 0.002) in the premenopausal group compared to the postmenopausal group, while there was a significant decrease in *HMGA2* expression (p value = 0.002). A positive correlation between patient age and *HMGA2* and a negative correlation between patient age and miR-20a-5p were found (respectively p value: 0.037 and p value: 0.006*)*. Also, we found a negative correlation between *HMGA2* and miR-20a-5p (p value: 0.027, correlation coefficient: −0.350).

**Conclusion:**

To our knowledge, this study is the first to examine the association of the *HMGA2* associated HOTAIR axis with breast cancer in cell-free RNA from peripheral blood of patients. Our findings emphasize the potential of the *HMGA2* associated HOTAIR axis as a prognostic biomarker and therapeutic target, especially in ER negative, postmenopausal onset, and relapsed breast cancer.

## 1. Introduction

Breast cancer is the most commonly diagnosed cancer worldwide, according to data from the 2020 Global Cancer Observatory (GLOBOCAN) [1]. Nonmodifiable risk factors include age, race, female sex, and genetic predisposition. The incidence peaks in the perimenopausal period, suggesting that hormonal mechanisms are also involved in its development [2].

Breast cancer is a heterogeneous cancer and is classified into different types Histopathologically, carcioma of the breast is divided into 2 main classes including invasive ductal carcinoma and invasive lobular carcinoma. In addition, there are more than ten rare subtypes [3]. Molecular classification is mostly used for prognosis and treatment of breast cancer and is classified into 4 subtypes: luminal A, luminal B, HER2-OE (overexpression), and basal-like. While luminal A and luminal B types have a relatively better prognosis, HER2-OE and basal-like breast cancers show a more aggressive clinical course [4].

Breast cancer development involves several genetic and epigenetic changes. Epigenetic alterations affect DNA function without changing the DNA sequence, unlike genetic mutations [5]. DNA methylation, histone modification, and noncoding RNA (ncRNA) interaction are some of the epigenetic factors. Noncoding RNA sequences are divided into 2 main categories based on their nucleotide length. Long RNA sequences are composed of lncRNA, while short RNA sequences consist of microRNA (miRNA), circular RNA (circRNA), and small nucleolar RNA (snoRNA) [6]. The noncoding RNA family is involved in carcinogenesis by disrupting various epigenetic mechanisms [7]. Several lncRNAs such as Hox Transcript Antisense Intergenic RNA (HOTAIR), Metastasis Associated Lung Adenocarcinoma Transcript 1 (MALAT1) and Maternally Expressed 3 (MEG3) can regulate gene expression by acting as competing endogenous RNA (ceRNA) to sponge microRNAs [8–10]. In summary, lncRNAs compete with miRNAs to affect shared target mRNAs [11]. The noncoding RNA TCL6 has been identified as a critical factor in the development of breast cancer, functioning through a ceRNA network of interactions involving miR-876-5p and the *MYL2* gene [12]. As reported in the literature, the ceRNA network-based prognostic model correlates with clinical outcomes, and the MAPK signaling pathway plays a critical role in the progression of triple negative breast cancer [13]. HOTAIR has been demonstrated to modulate the expression of downstream genes by engaging with a set of eight microRNAs (miRNAs). This ceRNA network has been shown to exert a significant impact on the clinical outcomes of patients diagnosed with breast cancer [14].

HOTAIR is one of the cancer-associated lncRNAs, many studies have shown that it is overexpressed in various solid tumors, in cancer initiation, progression, angiogenesis, and metastasis [15,16]. HOTAIR has also been reported to be involved in epigenetic mechanisms underlying breast cancer [17]. Its function is to act as a scaffold to epigenetically silence the expression of certain genes through epigenetic mechanisms. Additionally, its involved in regulating DNA functionality, including DNA methylation and histone modification [18].

MicroRNAs (miRNAs) are essential components of the regulatory network of gene expression. MiR-20a-5p expression has been reported to be dysregulated in various neoplasms, including breast cancer, pancreatic cancer, and nasopharyngeal cancer. HOTAIR functions as a ceRNA, thereby modulating the activity of miR-20a-5p within the regulatory network [19].

High mobility group protein 2 (HMGA2) regulates gene expression by binding to adenine-thymine (AT) dinucleotide-rich regions of DNA. While it is highly expressed in embryonic stem cells during embryogenesis, its expression level decreases significantly at later stages of development. However, an increase in expression levels has been observed in adults with cancer [20].

NcRNA levels show cancer-specific expression profiles during cancer development and progression. Cell-free RNAs can serve as biomarkers for cancer diagnosis and progression and that treatment regimens and targets can be developed by understanding their mechanisms of action. Effective treatment results can be achieved by targeting both genetic and epigenetic alterations in personalized cancer therapy [21,22].

This study aims to investigate the *HMGA2* associated HOTAIR pathway, which has been implicated in carcinogenesis in breast cancer patients, at the molecular level and to evaluate its clinical significance.

## 2. Materials and methods

The study group consisted of 40 female patients diagnosed with breast cancer who applied to the outpatient clinics of İstanbul University-Cerrahpaşa (IUC), Cerrahpasa Medical Faculty (CTF), Department of Medical Genetics in 2022, and 10 healthy female volunteers were included in the control group. The study was conducted in the laboratories of the Genetic Diseases Diagnosis and Evaluation Centre (GETAM), IUC-CTF, in accordance with the Declaration of Helsinki. Institutional Review Board approved the study protocol with the decision number; date 04.04.2022/362671. Written informed consent was obtained from all participants.

### 2.1. Study group

Demographic data, risk factors, medical and family history, as well as the results of mammography, breast MRI, breast USG, and pathology reports were retrospectively obtained from the patients’ medical records.

Female patients with histologically diagnosed breast cancer, who had not received chemotherapy, radiotherapy, immunotherapy, or targeted therapy in the past 3 years, were included in the study. Patients were not included if they had a recurrence in less than the previous 3 years, or if they had a primary cancer in another part of the body at the time of diagnosis. The reason for excluding patients with relapse before 3 years was to rule out possible residual disease. The 3 relapsed patients included in the study were not diagnosed with cancer for 3 years after recovery. A receptor positivity of 15% was considered the threshold for ER status. The HER2 receptor status was determined based on fluorescence in situ hybridization (FISH) results.

### 2.2. Plasma elution and total RNA isolation

Two cc of peripheral blood from each participant were collected in EDTA tubes, and plasma was obtained within 2 h. To obtain plasma, it was centrifuged at 3000 rpm for 10 min and at 14,000 g for 10 min to remove cell debris. The resulting sample was aliquoted and stored at −80 °C until used.

Frozen plasma was thawed at room temperature before total RNA isolation. Total RNA was isolated using the miRNeasy Serum/Plasma Advanced Kit (QIAGEN, Germany) according to the kit instructions. The concentration, quality, and purity of the RNA samples were measured by NanoDrop 2000/2000c spectrophotometer (Thermo Scientific Inc). Each RNA sample was equalised to 25–35 nanograms for both cDNA synthesis.

### 2.3. cDNA synthesis

The miRCURY LNA RT Kit (QIAGEN, Germany) was used for cDNA synthesis from miRNA and lncRNA using template RNA. The RT2 First Strand Kit (QIAGEN, Germany) was used for cDNA synthesis from mRNA according to manufecturer’s protocol.

### 2.4. Real-time PCR

miRCURY LNA Sybr Green Mastermix Kit (QIAGEN, Germany) was used for real-time PCR of the HOTAIR and miR-20a-5p genes according to the kit protocol. Primer sequences are demonstrated in Table 1. For relative quantification of HOTAIR and miR-20a-5p gene expression data, U6-sno gene was used as an internal control.

RT2 Sybr Green Mastermix Kit (QIAGEN, Germany) was used for real-time PCR of the *HMGA2* gene according to the kit protocol. The *ACTB* (β-actinin) gene was used as an internal control gene for relative quantification of *HMGA2* gene expression data (Table 1). The PCR mixture was loaded into the Rotor Gene (QIAGEN, Germany) PCR device on a 72’ platform.

Melting curve analysis was performed after the RT-PCR study. The amount of gene expression was determined according to delta-delta Ct method and change in the expression was determined using the 2^−ΔΔCt^ method.

### 2.5. Statistical analysis

Demographic and clinical data of the study group were analysed using SPSS program (version 26.0 for Windows, SPSS Inc, Chicago, IL). The Mann-Whitney U test, a nonparametric test, was used to analyse expression changes between the study and control groups. Patients were grouped according to stage, menopausal status, recurrence, estrogen and progesterone receptor positivity, and HER2 status. The Mann-Whitney U test was used for analysis between groups. P value of <0.05 was considered statistically significant.

The Spearman’s Rho correlation coefficient was used for correlation analysis of miRNA, lncRNA, mRNA expressions, and age of patients. A p-value of <0.05 was considered statistically significant.

## 3. Results

Age and clinicopathological information of the patients are demonstrated in Table 2. Hormone receptor and HER2 receptor status of five patients could not be determined due to a lack of sufficient data. The mean rank, sum of rank, and p-values of HOTAIR, miR-20a-5p and *HMGA2* expressions are summarised in Table 3.

**Figure 1:**
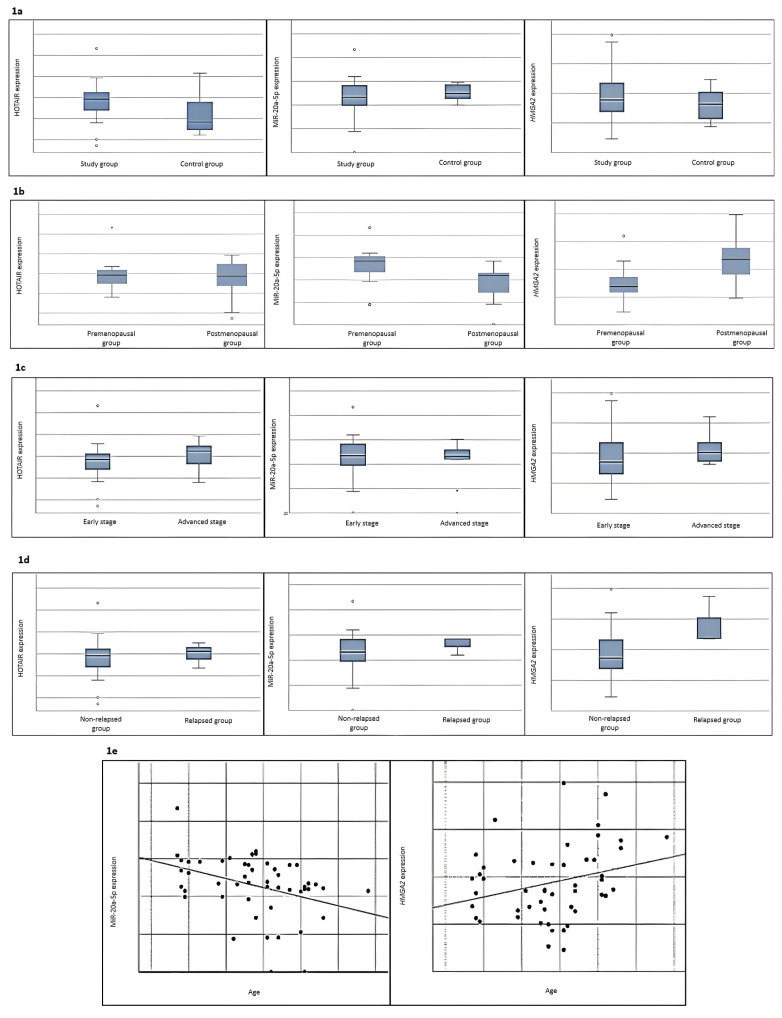
Figure 1a shows that HOTAIR expression was higher in the study group compared to the control group. Figure 1b shows that the expression of *HMGA2* were upregulated, while the expression of miR-20a-5p were downregulated in the postmenopausal group compared to the premenopausal group. As illustrated in Figure 1d, relapsed patients had higher levels of *HMGA2* expression. Furthermore, Figure 1e shows that *HMGA2* expression levels increased and miR-20a-5p expression levels decreased with increasing age.

First, expressions of HOTAIR/miR20a-5p/*HMGA2* genes were quantified and compared in breast cancer patients (n = 40) and healthy controls (n = 10). Expression of HOTAIR was upregulated in patients compared to controls (p value: 0.025). However, there was no difference in the expressions of miR-20a-5p and *HMGA2*, demonstrated in Figure 1a.

When we compared the premenopausal and postmenopausal groups, the expression of *HMGA2* were upregulated, while the expression of miR-20a-5p were downregulated in the postmenopausal group compared to the premenopausal group (p values: 0.002). As shown in Figure 1b, no significant difference was found for HOTAIR expressions. The expressions of HOTAIR and *HMGA2* were higher in late stage breast cancer patients than in early stage, as shown in Figure 1c.

*HMGA2* expression levels were also found to be statistically significantly higher in the relapsed group than in the nonrelapsed group (p value: 0.048). No significant difference was found for HOTAIR and miR-20a-5p expressions, as shown in Figure 1d.

**Figure 2:**
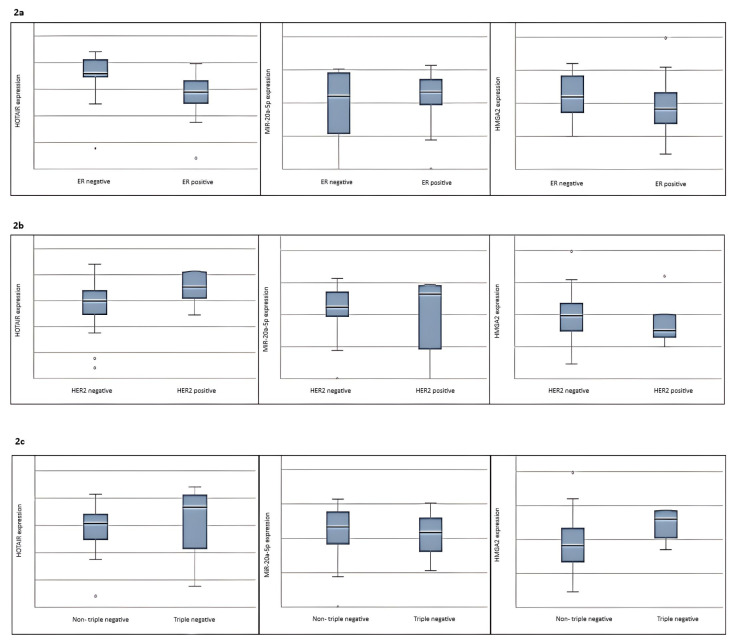
Figure 2a shows that expression of HOTAIR was upregulated in the ER-negative group compared to the ER-positive group. No significant difference was found when patients were compared for HER2-OE and Triple negative groups in all target RNAs. However, expression levels of HOTAIR were higher in the HER2-OE group, as shown in Figure 2b. In addition, Figure 2c presents the expression of *HMGA2*, which was higher in the triple-negative group.

When the study group was compared by ER status, expression of HOTAIR was upregulated in the ER-negative group compared to the ER-positive group. In contrast, both cohorts had similar expression levels of miR-20a-5p and *HMGA2*, as shown in Figure 2a.

No significant difference was found when patients were compared for HER2 receptor status in all target RNAs. However, expression levels of HOTAIR were higher in the HER2-OE group, as shown in Figure 2b. In addition, Figure 2c presents that the expression of *HMGA2* was higher in the triple-negative group.

**Figure 3:**
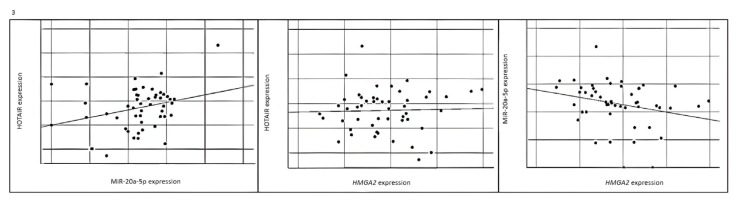
A statistically significant negative correlation was found between miR20a-5p and its target gene, *HMGA2*, as shown in Figure 3.

When we compared the correlation coefficient, a statistically significant negative correlation was found between miR20a-5p and its target gene, *HMGA2*, as shown in Figure 3. The p-value of the analysis was 0.027, and the correlation coefficient was −0.35. It was also found that *HMGA2* expression levels increased and miR-20a-5p expression levels decreased with increasing age (respectively p value: 0.037 and p value: 0.006), as shown in Figure 1e.

## 4. Discussion

Current research is focused on studying the epigenetic changes and regulatory functions of the noncoding RNA family [23,24]. LncRNAs on gene expression either post-transcriptionally or transcriptionally. They exert their effects through base pairing with miRNAs, enhancer regions of genes, or histone modification [25,26]. Recent studies have shown that HOTAIR facilitates protein-protein interactions influencing various pathways such as epigenetic reorganization, protein stability, and signal transduction in cancer [16,27]. MiR-20a-5p, which interacts with HOTAIR [19], has been evaluated as a biomarker in cervical cancer, breast cancer, and leukemia [28]. HOTAIR also affects the expression of *HMGA2*, an oncomir, through miR-20a-5p sponging [29]. Also, HOTAIR Although *HMGA2* is highly expressed in most malignant tumors, including ovarian and pancreatic cancer [30,31], there are limited studies on its association with tumor formation and progression in breast cancer.

We found that the expression of HOTAIR was higher in breast cancer patients than in controls (p value: 0.025), which is consistent with previous literature [17,32]. Dysregulated HOTAIR expression has been shown to play a role in tumor initiation, growth, angiogenesis, cancer progression, recurrence, drug resistance, and poor prognosis in most solid cancers [15,16,18].

We found that HOTAIR expression was statistically significantly higher in ER-negative patients compared to ER-positive patients (p value = 0.006). In a retrospective clinical study, HOTAIR overexpression in estrogen receptor positive (ER+) breast cancer patients was strongly associated with metastatic risk, suggesting that it may be a potential prognostic biomarker [33]. Another study showed that HOTAIR expression was increased in ER-cancers and was associated with poor prognosis [34]. Our results show that HOTAIR may be involved in the subtype-specific diagnostic process and prognosis determination, especially in ER-negative breast cancer patients.

Expression of HOTAIR was upregulated in patients with the HER2-OE subtype. However, the difference was not statistically significant. *HOTAIR* gene expression has been reported to correlate with HER2 positivity [35,36]. Liu et al. reported that ceRNA HOTAIR has an effect on *HER2* gene expression through miR-331-3p sponging [37]. The 2019 study also reported that circulating HOTAIR may be a sensitive fluid biomarker in patients with HER2-positive breast cancer [36].

MiR-20a-5p has been reported to act as a tumor suppressor in various cancers, such as leukemia, breast, and cervical carcinoma. Additionally, studies have shown that changes in miR-20a-5p expression levels can affect the PI3K-Akt, MAPK, and TGF-β signalling pathways [28]. Although it has tumor suppressor activity, it can cause cell proliferation and migration by targeting *RUNX3* in triple negative breast and cervical cancers [38,39]. On the contrary, there are conflicting reports about miR-20a-5p and its neoplastic role. It has been found to have an antineoplastic role in endometrial cancer through effects on Jak1 and STAT3 signaling [40]. Han et al. (2021) reported a decrease in the expression of miR-20a-5p in nonsmall cell lung cancer (NSCLC) tissues. They found that miR-20a plays a tumor suppressor role by affecting the PI3K/Akt pathway. The researchers also discovered that increased miR-20a expression suppressed NSCLC cell proliferation and endothelial migration in vitro and inhibited tumor growth and angiogenesis in vivo. Another study has reported that miR-20a-5p interacts with the lncRNA HOTAIR contributing to adriamycin resistance in patients with acute myeloid leukemia [27]. As indicated in the extant literature, miR-20a-5p has been reported to contribute to the development of breast cancer by affecting the regulation of *SPRy4-IT1*, *SRCIN1*, and *PANDAR* gene expression [28]. However, in our study, miR-20a-5p did not significantly correlate with different clinicopathologic groups of breast cancers. This might be due to its ability to promote or inhibit target pathways or to the small number of study group.

Xu et al. (2021) reported an increase in *HMGA2* gene expression in triple negative breast cancer (TNBC) cells [41]. Another found that *HMGA2* supports oncogenicity in TNBC. Therefore, *HMGA2* suppression could be an important therapeutic target as it reduces cancer cell proliferation, invasion, and migration. According to Mansoori et al. (2021), there was no significant increase in expression in luminal type and HER2 positive types [19]. Jun et al. (2015) identified *HMGA1* and *HMGA2* expressions as prognostic biomarkers for tumor recurrence of gastric cancer in their study [42]. The expression of *HMGA2* was higher in the triple-negative group and close to statistically significant (p value: 0.116). It was determined that there was no statistically significant difference in the HER2-OE, and ER+ subtypes in terms of *HMGA2* expressions. However, the significantly higher gene expression in the relapse group of our study suggests that *HMGA2* could serve as a biomarker for monitoring breast cancer relapse. This requires further support from functional studies and a larger sample size. HOTAIR and *HMGA2* expression levels were higher in the late stage group compared to the early stage group (respectively p value: 0.158 and 0.175). In the literature, both *HMGA2* and HOTAIR have been revealed to be effective in breast cancer development, progression, and metastasis [43,44].

However, in our study, a positive correlation was found between *HMGA2* expression and age, while a negative correlation was found between miR-20a-5p levels and age. The *HMGA2* gene expression is high during the embryonic and fetal period, but declines with age. Studies evaluating cancer patients have shown no significant relationship with age [45,46]. Also, we found that *HMGA2* expression was upregulated in the postmenopausal group compared to the premenopausal group. To the best of our knowledge, the literature has not previously reported the association of *HMGA2* and miR-20a-5p with menopausal status in breast cancer. We found a negative correlation between *HMGA2* and miR-20a-5p, which is believed to be the result of miRNA-target gene interaction.

Several studies have reported that HOTAIR functions as a competitive endogenous RNA. In nonsmall cell lung cancer, HOTAIR promotes cell growth, invasion, and migration by sponging miR-149-5p [47]. Cai et al. (2017) demonstrated that HOTAIR affects the NOTCH3 signaling pathway by sponging miR-613 in pancreatic cancer [48]. In 2017, Luan et al. study reported that increased expression of HOTAIR in malignant melanoma cells leads to progression by sponging miR-152-5p [49]. However, we did not find a correlation between HOTAIR and miR-2a-5p in our study.

HOTAIR has been identified as a promising biomolecule for noninvasive prognostic biomarker and targeted therapies in breast cancer [50]. In vivo and in vitro studies in breast cancer have shown that antisense oligonucleotides targeting HOTAIR are effective in reducing tumor volume. A recent study reported that soy derived isoflavones are potent modulators of multiple signaling pathways, including HOTAIR, in cancer prevention and treatment [51]. The potential of circulating biomarkers such as ctDNA, cell free RNA in the diagnosis, prognosis, and treatment monitoring of breast cancer is becoming increasingly important [52,53].

In conclusion, our study revealed a significant increase in HOTAIR expression, particularly in ER-negative breast cancer patients, when evaluating target RNA expression in relation to clinicopathologic findings. Additionally, we observed a statistically significant increase in *HMGA2* expression in the relapse group and postmenopausal group. Our analysis also revealed a significant negative correlation between *HMGA2* and miR-20a-5p. To our knowledge, this study is the first to examine the association of the *HMGA2* associated HOTAIR axis with breast cancer in cell-free RNA from peripheral blood of patients. Further studies are required to demonstrate the role of HOTAIR ceRNA network in the clinical course of breast cancer.

The limitations of the study are attributed to two primary factors: the restricted sample size of the study group, which was constrained by financial limitations, and the inability to generalise the findings to a broader population due to the small size of the study group. Additionally, this study lacks functional studies validating the molecular interactions between HOTAIR, miR-20a-5p, and *HMGA2*. In order to confirm these interactions, further experimental studies are required. Also, this is a correlational study and does not reveal a cause-and-effect relationship.

## Figures and Tables

**Table 1:** Target and control gene primer sequences.

**HOTAIR**	**Forward**	CAGTGGGGAACTCTGACTCG
	**Reverse**	GTGCCTGGTGCTCTCTTACC

**miR-20a-5p**	**Forward**	UAAAGUGCUUAUAGUGCAGGUAG
	**Reverse**	CUACCUGCACUAUAAGCACUUUA

**U6 (Control)**	**Forward**	CTCGCTTCGGCAGCACATATACT
	**Reverse**	ACGCTTCACGAATTTGCGTGTC

***HMGA2***	**Forward**	GGGCGCCGACATTCAAT
	**Reverse**	ACTGCAGTGTCTTCTCCCTTCAA

***ACTB*** ** (Control)**	**Forward**	CATGTACGTTGCTATCCAGGC
	**Reverse**	CTCCTTAATGTCACGCACGAT

**Table 2:** Clinicopathologic information of the patients.

Parameters	Number (percent)

**Patients number**	40 (100)

**Mean age Stage**	50.60 (±11.073)

Early stage	29 (72.5)

Advanced stage	11 (27.5)
**Diagnose**	
IDC	29 (72.5)
ILC	3 (7.5)
Others	8 (20)

**Grade**	
G1–G2	23 (69.7)
G3	10 (30.3)

**ER Status**	
Positive	25 (71.4)
Negative	10 (28.6)
**HER2-OE**	
Positive	6 (17.1)
Negative	29 (82.9)
**Triple-negative**	
Triple negative	4 (11.4)
Nontriple negative	31 (88.6)
**Relapse**	
Relapsed	3 (7.5)
Nonrelapsed	37 (92.5)

***Abbreviations:* (**IDC) Invasive ductal cancer, (ILC) Invasive lobular cancer.

Hormone receptor and HER2 receptor status of five patients could not be determined due to lack of sufficient data.

**Table 3:** The mean rank, sum of rank and p values of HOTAIR, miR-20a-5p and *HMGA2* expressions.

		HOTAIR			MiR-20a-5p			*HMGA2*	

	N	MR/SR	p-value	N	MR/SR	p-value	N	MR/SR	p-value

**Study group**	39	27.29 /1064.50	**p = 0.025** *	38	23.93/909.50	p = 0.591	40	26.78/1071.00	p = 0.224
**Control group**	10	16.05/160.50		10	26.65/266.50		10	20.40/204.00	

**Premenopausal**	27	24.41/659.00	p = 0.748	27	30.09/812.50	**p = 0.002** *	27	19.56/528.00	**p = 0.002** *
**Postmenopausal**	22	25.73/566.00		21	17.31/363.50		23	32.48/747.00	

**Early stage**	28	18.38/514.50	p = 0.158	28	19.45/544.50	p = 0.961	29	18.93/549.00	p = 0.175
**Late stage**	11	24.14/265.50		10	19.65/196.50		11	27.64/271.00	

**IDC**	28	20.48/573.50	p = 0.678	27	18.70/505.00	p = 0.505	29	21.14/613.00	p = 0.591
**Others**	11	18.77/206.50		11	21.45/236.00		11	18.82/207.00	

**ER positive**	24	14.60/350.50	**p = 0.006** *	24	16.96/407.00	p = 0.984	25	16.64/416.00	p = 0.225
**ER negative**	10	24.45/244.50		9	17.11/154.00		10	21.40/214.00	

**HER2-OE**	6	23.58/141.50	p = 0.100	5	21.00/105.00	p = 0.338	6	14.67/88.00	p = 0.403
**Others**	28	16.20/453.50		28	16.29/456.00		29	18.69/542.00	

**Triple-negative**	4	22.75/91.00	p = 0.285	4	14.25/57.00	p = 0.576	4	25.75/103.00	p = 0.116
**Others**	30	16.80/504.00		29	17.38/504.00		31	17.00/527.00	

**Relapse**	3	22.17/66.50	p = 0.746	3	23.00/69.00	p = 0.607	3	33.33/100.00	**p = 0.048** *
**Others**	36	19.82/713.50		35	19.20/672.00		37	19.46/720.00	

**Correlation parameters**				**Correlation coefficient**			**p-value**		

**Age and miR-20a-5p**				**−0.391**			**p = 0.006** *		

**Age and ** ***HMGA2***				**0.296**			**p = 0.037** *		

***HMGA2*** ** and miR-20a-5p**				**−0.350**			**p = 0.027** *		

**HOTAIR and miR-20a-5p**				0.230			p = 0.153		

**HOTAIR and ** ***HMGA2***				0.090			p= 0.582		

* p value of <0.05 was considered statistically significant.

***Abbreviations:*** (N) Number, (MR) Mean rank, (SR) Sum of rank, (IDC) Invasive ductal cancer, (ER) Estrogen receptor, (HER2-OE) HER2-over expression.
